# Phylogenetic Inferences and Historical Biogeography of Onocleaceae

**DOI:** 10.3390/plants14040510

**Published:** 2025-02-07

**Authors:** Jing Zhao, Jia-Guan Wang, Yu-Ping Hu, Chuan-Jie Huang, Shao-Li Fang, Zi-Yue Wan, Rong-Juan Li, Hong Yu, Zhao-Rong He, Xin-Mao Zhou

**Affiliations:** 1 School of Ecology and Environmental Science, Yunnan University, Kunming 650504, China; zhaojing@mail.ynu.edu.cn (J.Z.); huyuping@itc.ynu.edu.cn (Y.-P.H.); huangchuanjie@itc.ynu.edu.cn (C.-J.H.); sl@mail.ynu.edu.cn (S.-L.F.); wanziyue2000@outlook.com (Z.-Y.W.); lirongjuan@stu.ynu.edu.cn (R.-J.L.); hongyu@ynu.edu.cn (H.Y.); 2 School of Life Sciences, Yunnan University, East Outer Ring Road, Chenggong District, Kunming 650500, China; wangjiaguan@ynu.edu.cn

**Keywords:** biogeography, disjunction, phylogenetic, pteridophytes, long-distance dispersal

## Abstract

The family Onocleaceae represents a small family of terrestrial ferns, with four genera and around five species. It has a circumboreal to north temperate distribution, and exhibits a disjunct distribution between Eurasia and North America, including Mexico. Historically, the taxonomy and classification of this family has been subject to debate and contention among scholars, leading to contradictory classifications and disagreements on the number of genera and species within the family. Furthermore, due to this disjunct intercontinental distribution and the lack of detailed study across its wide range, this family merits further study to clarify its distributional pattern. Maximum likelihood and Bayesian phylogenetic reconstructions were based on a concatenated sequence dataset for 17 plastid loci and one nuclear locus, which were generated from 106 ingroup and six outgroup taxa from three families. Phylogenetic analyses support that Onocleaceae is composed of four main clades, and *Pentarhizidium* was recovered as the first branching lineages in Onocleaceae. Molecular dating and ancestral area reconstruction analyses suggest that the stem group of Onocleaceae originated in Late Cretaceous, with subsequent diversification and establishment of the genera *Matteuccia*, *Onoclea*, *Onocleopsis*, and *Pentarhizidium* during the Paleogene and Neogene. The ancestors of *Matteuccia*, *Onoclea*, and *Onocleopsis* could have migrated to North America via the Beringian land bridge or North Atlantic land bridge which suggests that the diversification of *Matteuccia* + *Onoclea* + *Onocleopsis* closely aligns with the Paleocene-Eocene Thermal Maximum (PETM). In addition, these results suggest that Onocleaceae species diversity peaks during the late Neogene to Quaternary. Studies such as this enhance our understanding of the mechanisms and climatic conditions shaping disjunct distribution in ferns and lycophytes of eastern Asia, North America, and Mexico and contribute to a growing body of evidence from other taxa, to advance our understanding of the origins and migration of plants across continents.

## 1. Introduction

The onocleoid ferns form a distinct group distinguished morphologically by rhizomes long- to short-creeping to ascending, sometimes stoloniferous, having dimorphic leaves, petioles with two vascular bundles, and thickened petiole bases, sori enclosed (sometimes tightly) by reflexed laminar margins, also with membranous, often fugacious true indusia, and chlorophyllous spores [1,2,3]. Onocleaceae Pic.Serm. is a lineage of terrestrial ferns with few species [2,3], which are generally accepted to include four genera (*Matteuccia* Tod., *Onoclea* L., *Onocleopsis* F.Ballard, *Pentarhizidium* Hayata) and about five species (e.g., *Matteuccia struthiopteris* (L.) Tod., *Onocleopsis hintonii* F.Ballard, *Onoclea sensibilis* L., *Pentarhizidium intermedium* (C.Chr.) Hayata, *Pentarhizidium orientale* (Hook.) Hayata; [4]). Although the species delimitation of Onocleaceae has rarely been controversial [5,6,7,8,9], the generic and infraspecific classification within Onocleaceae has been notoriously contentious [2,4,5,6,7,8,10,11,12,13].

Copeland [5], Llyod [6], and Tryon and Tryon [7] recognized that Onocleaceae consisted of three genera (*Matteuccia*, *Onocleopsis,* and *Onoclea*) and five species (*Matteuccia struthiopteris*, *M. intermedia* C.Chr., *M. orientalis* (Hook.) Trevis., *Onocleopsis hintonii*, *Onoclea sensibilis*). Kato and Sahashi [10] established a bi-generic (*Matteuccia* and *Onoclea*) classification of Onocleaceae, where each genus is subdivided into two sections (*M*. sect. *Matteuccia* [*M. struthiopteris*, *M. intermedia*], *M*. sect. *Onocleopsis* [*M*. *hintonii* (F.Ballard) M.Kato]; *O*. sect. *Onoclea* [*O. sensibilis*, *O. sensibilis* var. *interrupta* Maxim.], *O*. sect. *Pentarhizidium* [*O*. *orientalis* (Hook.) Hook.]). However, it should be noted that Kato and his collaborators have twice circumscribed the species of *Matteuccia* (*M. orientalis*, *M. intermedia*) into *Onoclea* (*O*. *orientalis*, *O. intermedia* (C.Chr.) M.Kato) and later on re-circumscribed these taxa at the rank of variety where *O*. *sensibiliis* is recognized as a North American endemic, and *O*. *sensibilis* var. *interrupta* as an East Asian endemic [10,11]. Kramer [8] returned to the tri-generic classification system of Copeland [5] and five-species classification of Onocleaceae, but diverged by recognizing that *Onoclea* is composed of only two sections (*O*. sect. *Onoclea* and *O*. sect. *Pentarhizidium*) and by placing *Matteuccia intermedia* into *Onoclea* sect. *Pentarhizidium*.

Gastony and Ungerer [1] first integrated nucleotide sequences of the plastid *rbc*L gene to infer the phylogenetic relationship of Onocleaceae based on 10 samples, which included all five species. Due to the limited number of informative sites in plastid markers, such as *rbc*L, the phylogenetic relationships between species have not been well supported (Maximum Parsimony Bootstrap Support [MP-BS] = 71–100; Maximum-Likelihood Bootstrap Support [ML-BS] = 67–100). Gastony and Ungerer [1] proposed two alternative classifications of Onocleaceae: (1) Four genera (*Matteuccia* [*M. struthiopteris*], *Onoclea* [*O. sensibilis*, *O. sensibilis* var. *interrupta*], *Onocleopsis* [*O. hintonii*], *Pentarhizidium* [*P. intermedium* (C.Chr.) Hayata, *P. orientale* (Hook.) Hayata]); (2) Three genera (*Matteuccia* [but divided into two sections, *M*. sect. *Matteuccia* (*M. struthiopteris*), *M*. sect. *Onocleopsis* [*M*. *hintonii*], *Onoclea* [*O. sensibilis*, *O. sensibilis* var. *interrupta*], and *Pentarhizidium* [*P. intermedium*, *P. orientale*]). More recently, Christenhusz et al. [13] circumscribed the family as a single genus *Onoclea*, but most pteridologists disagree with this circumscription, and recognize the four genera system as the accepted classification [2,3,4,12,14,15,16]. Following the study of Gastony and Ungerer [1], up to five accessions, and as many as five plastid makers, have been used to infer the phylogenetic relationship of Onocleaceae [14]. However, further research such as plastomes (chloroplast genomes), single-molecule sequencing, transcriptome sequencing, whole genome sequencing, and so on, have yet to confirm the phylogenetic relationships between species in the family Onocleaceae across their vast intercontinental ranges.

Another aspect warranting more detailed study in Onocleaceae concern its origins and disjunct distribution patterns. Onocleaceae is composed of five species largely distributed in north-temperate regions and Mexico [2,16,17]. *Pentarhizidium* contains two species and is the only genus in the family endemic to eastern Asia [3,17]. The most widely distributed species is *Matteuccia struthiopteris*, which is circumboreal in the northern hemisphere near river banks and is found floodplain forests [18]. *Onocleopsis hintonii* is a rare species and endemic to wet mountain canyons in southern Mexico and Guatemala [19]. *Onoclea sensibilis* is widely distributed in northern Asia and throughout central and eastern North America, where it is common near marshes, lakes, moist woodlands and other riparian environments [20]. *Onoclea sensibilis* has one of the most familiar and well-documented disjunct distribution between East Asian and North American fern species [16,21,22,23]. A vicariance hypothesis to explain this disjunct distribution has been proposed and is supported by fossil evidence which found that the *O. sensiblis* group was widely distributed in Alaska, Canada, the United States, Europe, and Japan in the Cretaceous and Tertiary [24], but it is suspected that long-distance dispersal (LDD) may also played a role in the history of this group [25].

Furthermore, the backbone support for species relationships remain poorly supported in this family [1]. Molecular dating analyses might be help to further clarify this point. The species level relationships and extant distributions in the family Onocleaceae merit further study in several areas. First, there is a need to determine for whether the disjunct populations of *Onoclea sensibilisis* should be treated as distinctly different species. Previous studies had suspected that *O*. *sensibilis* from Asian and North America were shown to be phylogenetically differentiated from each other, with only 4–7 bp different in *rbc*L [1]. Additionally, there is still outstanding disagreement on whether the extinct species, such as *O*. *fecunda*, are the same species as the extant species *O*. *sensibils* [26,27]. Other researchers have identified fossil evidence as extinct species, such as *O. fecunda* [28], *O. hebridica* [29], and *O. hesperia* [28]. Moreover, there is a clearly understudied aspect of the family in general, and a need to perform an ancestral area reconstruction to test various historical hypotheses.

In this context, the present study aims to achieve the following goals: (1) Generate and annotate novel plastomes for the Onocleaceace species; (2) Perform a phylogenetic reconstruction for the family; (3) Propose a biogeographic hypothesis for the origins, dispersal, and migration to their extant distributions.

## 2. Materials and Methods

### 2.1. Taxon Sampling, DNA Extraction and Sequencing

In the present study, 12 specimens from different locations or populations were newly sampled and genome skimming data were generated for the following taxa: *Matteuccia struthiopteris*, *Onoclea sensibilis*, *O*. *sensibilis* var. *interrupta*, *Pentarhizidium orientale*, *P*. *intermedium*, *Woodwardia japonica* (L.f.) Sm (Table 1 and Table S1). Total genomic DNA was extracted from silica-dried material using the TIANGEN plant genomic DNA extraction kit (TIANGEN Biotech., Beijing, China) following the manufacturers’ protocols. Sequencing was conducted on the Illumina NovaSeq 6000 platform at Biomaker Technology Co., Ltd. (Beijing, China) with paired-end sequence and an insert size of 350 bp. Voucher specimens for all the materials were deposited at PYU (Table 1 and Table S1), and 3.72 ± 0.62 Gbp of raw data were obtained for each sample.

### 2.2. Plastome Assembly, Annotation, and Phylogenetic Analysis

Sequencing adapters, reads containing Ns, and low-quality bases were removed with Fastp v0.12.4 [30] with default parameters. Plastid sequence reads were assembled using the software GetOrganelle v1.7.5 [31], with the reference plastid genomes of *Matteuccia struthiopteris* (NC035859; [32]). Each plastid genome was initially annotated using GeSeq [33] and CPGAVAS2 [34]. Start and stop codons of all loci were manually checked in Geneious Prime 2019.2.1. For any uncertain protein-coding genes, a Blastn search was performed with default parameter settings, and all tRNAs was verified using tRNAscan-SE v2.0 web server [35]. All the plastid genome sequences were deposited in GenBank (Table 1), circular gene map drawn by OmicsSuite v1.3.9 [36], and manually modified accordingly for clarity and accessibility. Additionally, the “embplant_nr” module was used to assemble Embryophyta plant nuclear ribosomal RNA (18S-ITS1-5.8S-ITS2-26S). In order to integrate all available public data, 13 plastid coding regions (*acc*D, *atp*A, *atp*B, *mat*K, *psb*A, *rbc*L, *rps*4, *trn*F, *trn*G, *trn*H, *trn*L, *trn*R, *trn*S), 4 plastid non-coding regions (*psb*A-*trn*H spacer, *rps*4-*trn*S spacer, *trn*G-*trn*R spacer, *trn*L-*trn*F spacer), and 1 nuclear maker (ITS) of 100 taxa were downloaded from GenBank, and the novel 18 loci were extracted and included for phylogenetic analyses. When necessary, five additional species belonging to Woodsiaceae, Athyriaceae, and Blechnaceae were chosen as outgroups [4,37]. In total, 112 taxa were included for sequent analyses (Table S2). Sequences were aligned with MAFFT v7.450 [38] implemented in Geneious Prime before concatenation. The alignments were manually assembled and edited using BioEdit v7.0.5.3 [39] for quality control and to remove ambiguous sites. To infer the appropriate nucleotide substitution model for the phylogenetic analyses, ModelFinder [40] was employed, and the model was selected based on the bias-corrected Akaike information criterion (AICc). A maximum likelihood (ML) tree was generated by performing a rapid bootstrap analysis on IQ-tree v2.1.3 [41] with the selected GTR + F + I + G4 model for both partitions. After 5000 rapid bootstrap search step, ML bootstrap values (ML-BS) from each node were visualized using FigTree v1.4.3 [42]. The Bayesian inference (BI) analysis was performed in MrBayes v3.2.7 [43] based on the model identified in the ModelFinder analysis, using one million generations with one tree sampled every one thousand generations; four runs with four chains were performed in parallel. The first 25% of trees were discarded as burn-in. The standard deviation of splits frequencies below 0.001, and the Markov Chain Monte Carlo (MCMC) output was examined to check for convergence and to ensure that all of the effective sample size (ESS) values were >200. Four chains were run, each for two million generations, and were sampled every one hundred generations, with a random starting tree. Bayesian posterior probabilities (BI-PP) were calculated for the majority consensus tree of all sampled trees after discarding trees sampled within the burn-in phase in MrBayes. In addition, we implemented two measures for quantifying genealogical concordance in concatenated datasets through IQ-tree: the gene concordance factor (gCF) and the site concordance factor (sCF) [44,45].

**Table 1:** Taxa and plastomes features overview used in this study.

Taxon	Plastome Size (bp)	GC Content (%)	LSC Size (bp)	LSC GC Content (%)	SSC Size (bp)	SSC GC Content (%)	IR Size (bp)	IR GC Content (%)	Voucher	Herbarium Acronyms	Location	GenBank ID	Reference
*Matteuccia struthiopteris* (L.) Tod.	151,101	44.2	81,974	43.7	21,695	42.1	23,716	46.1	YUS8847	PYU	China, Sichuan	PP712888	This study
*Matteuccia struthiopteris* (L.) Tod.	151,078	44.3	82,020	43.8	21,672	42.2	23,693	46.1	Wei Q. et al. FB854	KUN	China, Yunnan	MT130666	[37]
*Matteuccia struthiopteris* (L.) Tod.	151,003	44.3	81,964	43.8	21,675	42.2	23,682	46.1	WR0331	PE	China, Beijing	NC035859	[32]
*Onoclea sensibilis* L.	148,604	44.4	81,588	44.1	21,730	42.5	22,643	45.9	ZhouXM677	PYU	USA, Cult.	PP712887	This study
*Onoclea sensibilis* L.	148,395	44.4	81,571	44.1	21,726	42.6	22,549	45.8	WR0327	PE	China, Beijing	NC035860	[32]
*Onoclea sensibilis* var. *interrupta* Maxim.	138,259	44.7	75,518	44.3	17,057	42.9	22,903	45.9	ZhaoJingLN	PYU	China, Liaoning	PP712885	This study
*Onoclea sensibilis* var. *interrupta* Maxim.	141,826	44.6	77,871	44.3	19,089	42.9	22,039	45.9	ZhaoJingLN3	PYU	China, Liaoning	PP712886	This study
*Onoclea sensibilis* var. *interrupta* Maxim.	148,739	44.4	81,629	44.1	21,744	42.5	22,683	45.9	Lu J.M. Lu472	KUN	China, Jilin	MT130573	[37]
*Pentarhizidium intermedium* (C.Chr.) Hayata	151,168	44.2	82,554	43.7	21,620	42.2	23,497	46	YUS7829	PYU	China, Yunnan	PP712890	This study
*Pentarhizidium intermedium* (C.Chr.) Hayata	151,167	44.2	82,555	43.7	21,620	42.2	23,496	46	YUS7844	PYU	China, Yunnan	PP712889	This study
*Pentarhizidium intermedium* (C.Chr.) Hayata	151,175	44.2	82,562	43.7	21,619	42.2	23,497	46	YUS9511	PYU	China, Yunnan	PP712891	This study
*Pentarhizidium orientale* (Hook.) Hayata	151,279	44	82,646	43.5	21,591	41.9	23,521	45.9	YUS6905	PYU	China, Yunnan	PP712894	This study
*Pentarhizidium orientale* (Hook.) Hayata	151,333	44	82,628	43.5	21,591	41.9	23,557	45.9	YUS8029	PYU	China, Yunnan	PP712895	This study
*Pentarhizidium orientale* (Hook.) Hayata	151,203	44	82,571	43.5	21,590	41.9	23,521	45.9	YUS9702	PYU	China, Yunnan	PP712893	This study
*Pentarhizidium orientale* (Hook.) Hayata	151,202	44	82,570	43.5	21,590	41.9	23,521	45.9	YUS10377	PYU	China, Yunnan	PP712892	This study
*Pentarhizidium orientale* (Hook.) Hayata	151,243	44	82,610	43.5	21,591	41.9	23,521	45.9	Lu J.M. Lu715	KUN	China, Jiangxi	MT130641	[37]
*Woodwardia japonica* (L.f.) Sm.	153,708	43.2	82,377	42.4	21,559	40.5	24,886	45.8	YUS8839	PYU	China, Sichuan	PP712896	This study

### 2.3. Molecular Dating and Historical Biogeography

Following the guideline of Maurin [46], penalized likelihood dating analysis was undertaken in treePL v2.6.3 [47] using the same sequence partitions as in phylogenetic analyses. One thousand bootstrap replicates using the best ML tree as a topology constraint were conducted in IQ-tree. Randomly sampled cross-validation analysis for the best ML tree was conducted with rate-smoothing values from 10^10^ to 10^−30^ and a multistep of 0.1, which resulted in an optimal smoothing parameter. Then, the bootstrap trees were dated using the best smoothing values. The output trees were used to summarize the maximum clade credibility (MCC) tree and confidence interval using TreeAnnotator v2.6.3 [48]. Three fossil calibration points were employed in this study as the minimum and the maximum age constraints in the divergence time estimates: (1) a fossil of *Athyrium cretaceum* Chen et Meng as the stem age of Athyriaceae reported from Neocomian (Berriasian– Hauterivian) (129.4–145.0 Ma; [49]) was used recently in dating analyses [37]; (2) a fossil of Woodwardia (72.5–76.1 Ma; [50]) found in south-central New Mexico was used here to constrain the divergence between Blechnaceae and Onocleaceae, which also was used recently in dating analyses [37]; (3) a fossil of *Woodwardia gravida* Hickey reported from the late Palaeocene (55.4–56.8 Ma; [51]), which was used here as the stem node of Blechnaceae being extensively utilized in other fern and lycophyte molecular dating analyses (e.g., [37,52,53,54]).

Five continental and subcontinental regions were delineated based on the occurrence records of the extant taxa, including (A) South Asia, (B) East Asia, (C) Palearctic, (D) North America, and (E) Mexico. Ranges of extant taxa were determined from a survey of the literature [6,10,17,19,55,56], herbaria, field work, and online databases of GBIF (https://www.gbif.org; [57]), Tropicos (http://www.tropicos.org), and JSTOR (http://plants.jstor.org/). To infer the ancestral distribution within Onocleaceae, we used the chronogram resulting from treePL as the input file to perform an ancestral area reconstruction after removing outgroup taxa. We first tested the best-fitted models available in the R package BioGeoBEARS [58] by time stratification events. A dispersal multiplier matrix was specified following the definition of Buerki et al. [59] and Wei et al. [60]: low dispersal = 0.01; medium dispersal = 0.5; high dispersal = 1.0 (Table S3), and analyses were carried out with a distance matrix. We tested the six models (DEC, DEC + J, DIVALIKE, DIVALIKE + J, BAYAREALIKE, BAYAREALIKE + J; [61]) implemented in BioGeoBEARS, the model with the highest AICc weight value selected has the best fitting model, and the maximum area number was set to four. We also estimated the number and type of biogeographic events in RASP v4.4 [62]. After providing a biogeographical model, the stochastic mapping algorithm generates simulations across nodes and branches of the provided phylogeny [63], including the times and locations of all events along the branches in that simulation. Event frequencies were taken to be the mean of event counts from 50 simulations.

## 3. Results

### 3.1. Plastome Organization and Features

**Figure 1:**
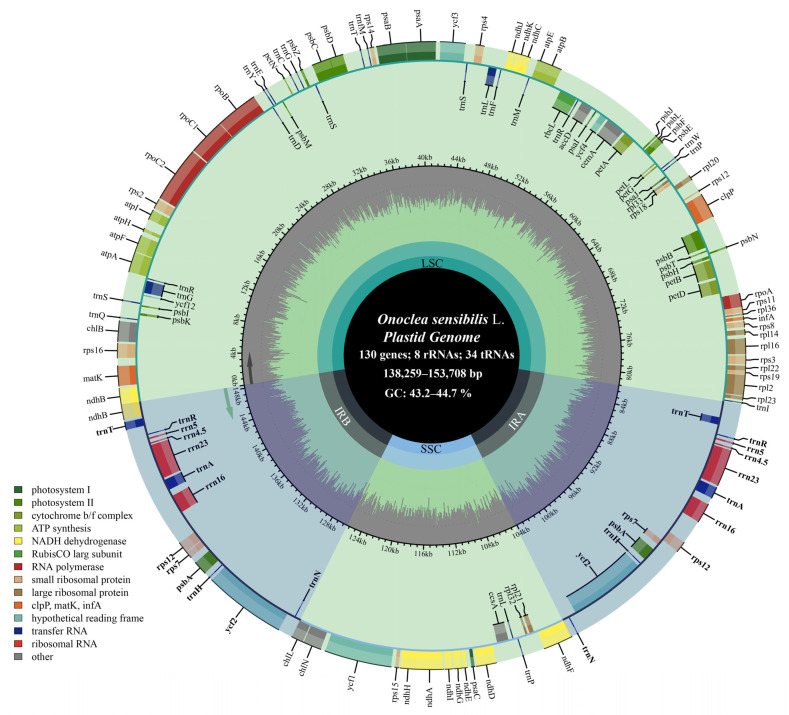
The plastome map of *Onoclea sensibilis* L. Green background represents single copy regions, blue background represents repeat regions. The dark gray track inside the map shows the GC content. Genes on the outside of the map are transcribed clockwise, and genes on the inside are transcribed counterclockwise. Genes belonging to different functional groups are shown in different colors; see the legend for groups. Bold indicated duplicated gene pairs.

The newly sequenced plastomes of the 12 individuals of Onocleaceae exhibited a typical quadripartite structure and included a large single-copy (LSC) region and a small single-copy (SSC) region separated by two inverted repeat (IR) copies (Figure 1). By integrating all complete plastomes of Onocleaceae available in GenBank (Table 1), the plastome size ranged from 138,259 bp in *Onoclea sensibilis* var. *interrupta* to 151,333 bp in *Pentarhizidium orientale*. The overall GC content ranged narrowly from 44.0% to 44.7%, whereas the GC content in the LSC, SSC, and IR regions varied from 43.5% to 44.3%, 41.9% to 42.9%, and 45.9% to 46.0%, respectively (Figure 1; Table 1). We observed only marginal variation in the IR length, which ranged from 22,039 bp in *O. sensibilis* var. *interrupta* to 23,716 bp in *Matteuccia struthiopteris* (Table 1). Substantial length variation was evident in the LSC and SSC, with LSC ranging from 75,518 bp in *O. sensibilis* var. *interrupta* to 82,646 bp in *Pentarhizidium orientale*, and SSC ranging from 17,057 bp in *O. sensibilis* var. *interrupta* to 21,695 bp in *M. struthiopteris* (Table 1).The plastomes of all 12 accessions of Onocleaceae encoded a set of 130 genes, of which 117 were single-copy and 13 were duplicated gene pairs in the IR regions (Figure 1). Among the 117 unique genes, there were 85 protein-coding genes, 28 tRNA genes, and four rRNA genes (Figure 1). Five tRNA genes and nine protein-coding genes contained a single intron, and three genes including *rps*12, *clp*P, and *ycf*3 contained two introns (Figure 1). The 5′-end exon of the *rps*12 gene was located in the LSC region, and the intron and 3′-end exon of the gene were situated in the IR region (Figure 1).

### 3.2. Phylogenetic Relationships

**Figure 2:**
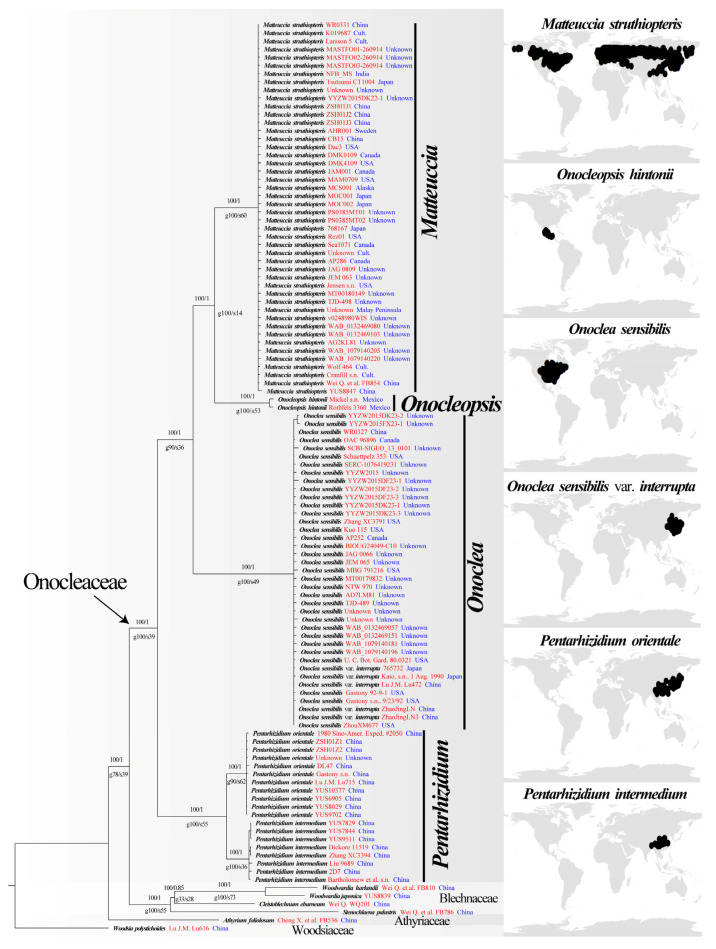
Phylogenetic tree reconstruction using Bayesian inference (BI) based on concatenated dataset. The numbers above the branches represent ML-BS/BI-PP. The numbers below the branches represent gCF/sCF. Voucher information and geographical provenance are indicated in red and blue, respectively. Maps show the distribution of species based on Global Biodiversity Information Facility (GBIF) data as black points for the species.

The BI tree of Onocleaceae reconstructed from the combined dataset are shown in Figure 2, and the dataset consists of the 13 plastid coding regions (*acc*D, *atp*A, *atp*B, *mat*K, *psb*A, *rbc*L, *rps*4, *trn*F, *trn*G, *trn*H, *trn*L, *trn*R, *trn*S), four plastid non-coding regions (*psb*A-*trn*H spacer, *rps*4-*trn*S spacer, *trn*G-*trn*R spacer, *trn*L-*trn*F spacer), and one nuclear gene marker (ITS) was 12,422 nucleotides in length.

The monophyly of Onocleaceae was strongly supported at the crown node with 100% ML-BS, a BI-PP of 1.00, 100% gCF, and 39% sCF (Figure 2). ML and BI analyses recovered four main clades, each supported by high statistical values (ML-BS and BI-PP; Figure 2). Almost all major branches within the ingroup were strongly supported (ML-BS = 100; BI-PP = 1.0; gCF = 90% to 100%; sCF = 36% to 62%; Figure 2). *Pentarhizidium* was recovered in a clade with two monophyletic species sisters to the rest of the family which are the only lineage endemic to Asia (Figure 2). *Onoclea* was recovered sister to a clade composed of the circumboreal *M*. *struthiopteris* and the Mexican endemic *Onocleopsis*. In addition, a monophyletic *Onoclea sensibilis* var. *interrputa* was not supported by the phylogenetic analysis and there was no clear geographic pattern based on the topology in the recovered phylogenetic inferences (Figure 2). The remaining lineages were composed of two monotypic genera (Figure 2), *Matteuccia struthiopteris* has a circumboreal distribution, while *Onocleopsis hintonii* is only found in the Neotropics (Table 2; Figure 2).

### 3.3. Biogeographic History and Ancestral Area Reconstruction

**Figure 3:**
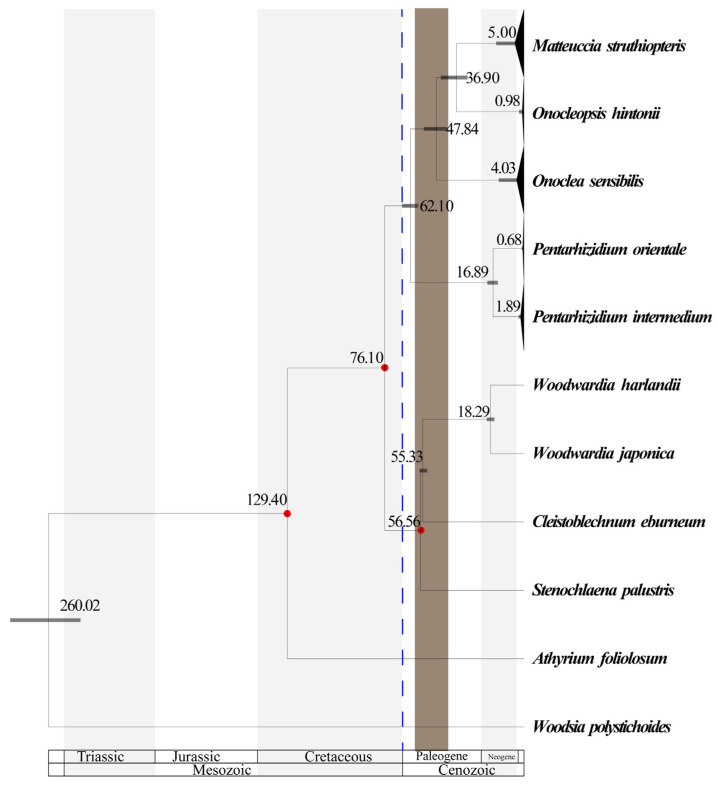
Time-calibrated phylogeny inferred by treePL based on concatenated dataset of Onocleaceae. The divergence times (Ma) are shown above the nodes. Black bars on nodes indicate the 95% highest probability density interval of the age. Fossil calibrated nodes are indicated by red dots. Blue dotted line indicates the K/Pg boundary. The brown stripe corresponds to the hottest period of the Cenozoic era (Paleocene-Eocene Thermal Maximum: PETM).

The molecular dating analysis estimated divergence between Onocleaceae and Blechnaceae to be 76 Ma (95% highest posterior density (HPD) ranged from 76.0 to 76.1 Ma; late Cretaceous; Figure 3). The crown of Onocleaceae diversified in the early Paleogene (95% HPD ranged from 57.9 to 66.7 Ma) with the subsequent establishment of the genera *Matteuccia*, *Onoclea*, *Onocleopsis*, and *Pentarhizidium* during the Paleogene and Neogene and these genera subsequently diversified from the late Neogene to the Quaternary (0.68 to 5 Ma; Figure 3). The estimated age for the split between *Onoclea* and *Matteuccia* + *Onocleopsis* was Eocene (95% HPD ranged from 42.0 to 54.7 Ma; Figure 3). The estimated age for the split between *Matteuccia* and *Onocleopsis* was between the Oligocene and the Eocene (95% HPD ranged from 30.8 to 45.5 Ma; Figure 3).

**Figure 4:**
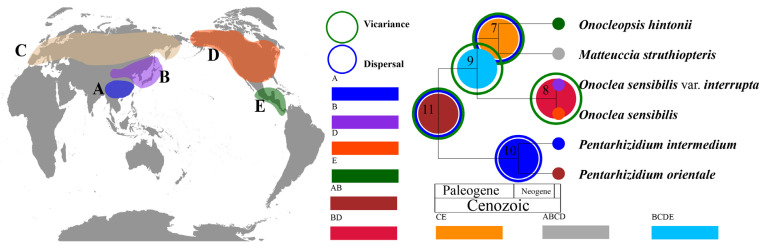
Ancestral range estimation used BioGeoBEARS to implement in RASP under the DIVALIKE model. The distribution of each species is mapped to the right of the chronogram. The single-most-probable state (geographical range) is shown at each node. The numbers above the branches represent node number. The green circle and blue circle around the nodes represent vicariance events and dispersal events, respectively.

The best fitting biogeographic model was DIVALIKE with the highest AICc weight value (Table 3). Asia was recovered as the most probable ancestral area (11.22%; Table S4) for the Onocleaceae crown node (node 11; Figure 4). For each of the three genera of *Matteuccia*, *Onoclea*, and *Onocleopsis*, we found evidence of Asian origins followed by migration to (C) Palearctic, (D) North America, and (E) Mexico. We detected three dispersal events and four vicariance events within Onocleaceae (Figure 4).

## 4. Discussion

### 4.1. Conservative Plastome Characteristics in Onocleaceae

In recent times, the growing availability of new plastome data from lycophytes and ferns has facilitated a clearer understanding of the evolutionary trends in structural variations within vascular plant plastomes [64,65,66,67]. Previous research works have established the observed patterns of plastome variation in ferns and lycophytes, encompassing inversions, shifts in IR boundaries, and alterations in gene content [68,69]. However, our results demonstrate that the gene number, gene order, and GC content of the plastid genome are largely consistent among eupolypods II and other polypod ferns [32,70,71,72]. Generally, we also supported that the plastome structure and gene content are highly conserved across Cretaceous to Cenozoic among members of the eupolypods II (Figure 1; Table 1; [32,66]).

### 4.2. Systematic Implications

The phylogenetic results of this study are largely in agreement with prior results that were based on a smaller sample of individuals [1,14]. Onocleaceae formed a well-supported clade (ML-BS = 100; BI-PP = 1.0; Figure 2). The inferred phylogenetic tree includes all six currently recognized taxa in the family, and strongly supports that this family contains four distinct evolutionary lineages (Figure 2). With the exception of the genus *Pentarhizidium*, which was recovered with two species, all other clades contained only one species (Figure 2 and Figure 3). It worth noting that *Matteuccia* recovered a sister to *Onoclea* based on nuclear loci, but *Matteuccia* recovered a sister to *Pentarhizidium* based on concatenated plastid locus (available at: doi.org/10.6084/m9.figshare.25203950, accessed on 11 November 2024), which implied there were might ancient gene flow [73,74] existed but need further study. In this study, *P. orientalis* and *P. intermedia* from Asia were recovered sister to the rest of the family with the other genera diversifying after *Pentarhizidium* had already diverged from the common ancestor to the family (Table 2; Figure 2, Figure 3 and Figure 4). Gastony and Ungerer [1] proposed to consider *Onoclea interrupta* (Maxim.) Gastony and Ungerer as a distinct species, endemic to eastern Asia and *O*. *sensibilis* as a species endemic to eastern North America. Maximowicz [75] recognized the Asian specimens as *O*. *sensibilis* var. *interrupta* Maxim based on the longer fertile frond, and sorus-bearing segments remote from each other. Our ML analysis also found that *O. sensibilis* formed two independent branches with weak support (available at: doi.org/10.6084/m9.figshare.25203950, accessed on 11 November 2024), but the BI analysis did not support that population of *O*. *sensibilis* from Asia to be sufficiently differentiated from North American populations to recognize *O*. *interrupta* at the species level (Figure 2). The specimens as *O. sensibilis* fossilis reported from Middle to late Paleocene (ca. 56 Ma), which might represent an extinct species or ancestor to the extant species [28,29]. In addition, molecular dating in this analysis recovered the stem age of extant *O. sensibilis* to have originated during the Paleogene around 47.84 Ma and the crown node of this species was recovered in the Pliocene (5 Ma; Figure 3). Furthermore, the four genera have been regarded as a single genus *Onoclea* [13]. We support Onocleaceae consists of four clades (Figure 2 and Figure 3; Table 2), and here advocate for the recognition of each clade at the genus rank in Onocleaceae based on morphological variation in addition to the results of the molecular dating and biogeographic analysis in this study. Molecular dating estimated that the stem ages of the four genera were dated back to the Paleogene, and at least four vicariance events were detected (Figure 4), which fall in the known range of previous studies using molecular based dating methods (e.g., [16,37,52,53,54,76,77]; Figure 3).

### 4.3. Origin and Historical Biogeography of Onocleaceae

Although the intercontinental disjunct distributions are well documented in fern resulting from the breakup of the Gondwanan supercontinents [76], the divergence times between the Onocleaceae and Blechnaceae lineages recovered in this study are too recent to be explained by a vicariance caused by the breakup of the Gondwana (120–140 Ma) [78]. Our results support an origin of crown Onocleaceae on continental Asia at around 62 Ma with both long-distance dispersal events and vicariance events to have occurred from continental Asia to other regions during the Paleogene (Figure 3 and Figure 4; Table S4). These results are consistent with the fossil record and previous studies using divergence time estimation to indicate that Onocleaceae originated during the Paleogene [54,76,79]. It is worth noting that thousands of fossils *Onoclea* specimens have been collected in the Paleocene sediments, and the molecular dating results (Figure 3) indicated that due to the Cretaceous Paleocene (K-Pg) extinction event, and the drastic environmental changes around the K-Pg boundary at that time led to the extinction of a large number of Onocleaceae [16,77,80,81,82]. Additionally, the abiotic and biotic factors after the K-Pg boundary may have facilitated the diversification of the other lineages within the family of Onocleaceae (Figure 3). Although *Pentahizidium* is now widely distributed in both (A) South Asia and (B) East Asia (Figure 4; Table S4), the ancestral area of *Pentarhizidium* was assumed that area (A) South Asia is most likely the original area (node 10; Figure 4; Table S4). The dispersal of *Pentarhizidium* from South Asia to East Asia could have happened after experiencing many rapid uplifts and unroofing of the Hymalayas. During this period (95% HPD ranged from 14.3 to 20.0 Ma; Figure 3), the collision between India and Asia (~22–25 Ma) resulted in the orogeny, which in turn altered the formation of landforms and climate zones in the region of Indochina [83]. Some fern genera, such as *Pyrrosia* [84] and *Platycerium* [85], have also been reported to have such patterns. The diversification of *Matteuccia* + *Onoclea* + *Onocleopsis* closely aligns with the Paleocene-Eocene Thermal Maximum (PETM), a period during which a well-documented boreotropical flora was extensively distributed in the high northern latitudes [86,87]. Our results show that intercontinental migration events in Onocleaceae occurred in the Eocene (36.9–47.8 Ma) (Figure 3 and Figure 4). The biogeographic analysis supports the ancestral range of the ancestor of *Matteuccia* + *Onoclea* + *Onocleopsis* to be widespread in the Northern Hemisphere (node 9; Figure 4; Table S4). The fossil record showed *Onoclea* was widely distributed in North America, Europe, and Japan in the Cretaceous and Tertiary [27] which supported the hypothesis that Onocleaceae once had a wider distribution. For *Onoclea*, paleogeographic changes and/or climatic cooling as well such as competition from other cold-adapted species during the Eocene had probably caused habitat reduction and extensive extinction of *Onoclea* species in the Northern Hemisphere. Starting from the early Eocene, global climate showed an overall cooling with a sharp temperature drop at the end of the Eocene termed the terminal Eocene event [88,89]. We hypothesize that the ancestors of *Matteuccia* + *Onoclea* + *Onocleopsis* could have migrated via the Beringian land bridge (BLB), which connected the two continents at least until the Quaternary, and the North Atlantic land bridge (NALB), which existed from the Late Cretaceous to the Paleogene [90,91]. The extant species of Onocleaceae have a higher species diversity during the late Neogene to Quaternary might be influenced by paleoclimate change ([16] Figure 3). Throughout the Neogene and Quaternary periods, climatic variations and major geological events also may have been influential in driving complex processes of migration/dispersal, extinction, and speciation, resulting in intercontinental disjunct distributions of plants in the Northern Hemisphere [92].

Taken together, our results enhance our understanding of the mechanisms and climatic conditions shaping disjunct distribution of ferns and lycophytes in eastern Asia, North America, and Mexico and contribute to a growing body of evidence from other taxa, advancing our understanding of the origins and migration of plants across continents.

## Figures and Tables

**Table 2:** Comparison of characters.

Genus	*Matteuccia*	*Onoclea*
Rhizomes	erect	creeping
Lamina	dimorphic	dimorphic
Trophophylls veins	open pinnate, free	reticulate
Chromosome number	x = 39, 40	x = 37
Glandular hairs of gametophytes	absent	present
Distribution	Disjunctly circumboreal	North America and Eastern Asia
**Genus**	***Onoclepsis***	***Pentarhizidium***
Rhizomes	erect	creeping
Lamina	dimorphic	dimorphic
Trophophylls veins	reticulate	open pinnate, free
Chromosome number	x = 40	x = 40, 41
Glandular hairs of gametophytes	absent	present
Distribution	Southern Mexico and Guatemala	Asiatic

**Table 3:** Results of the BioGeoBEARS analysis.

Model	LnL	Numparams	d	e	j	AICc	AICc_wt
DEC	−14.12	2	0.018	0.05	0	36.24	0.2
DEC + J	−13.04	3	0.013	0.037	0.23	44.07	0.004
DIVALIKE	−13.22	2	0.016	0.04	0	34.45	0.49
DIVALIKE + J	−12.89	3	0.015	0.045	0.28	43.77	0.0046
BAYAREALIKE	−13.71	2	0.54	2.38	0	35.41	0.3
BAYAREALIKE + J	−13.67	3	0.56	2.35	0.96	45.33	0.0021

## Data Availability

The multiple sequence alignments, concatenated alignments, and phylogenetic trees for this study are publicly available at Figshare repository: https://doi.org/10.6084/m9.figshare.25203950, accessed on 11 November 2024.

## Supplementary Materials

The following supporting information can be downloaded at: https://www.mdpi.com/article/10.3390/plants14040510/s1, Table S1: Collect information of taxa newly sampled; Table S2: List of taxa sampled with information related to taxonomy, GenBank accession numbers, references, and voucher information; Table S3: Dispersal matrix. Five continental and subcontinental regions were delineated: (A) South Asia, (B) East Asia, (C) Palearctic, (D) North America, and (E) Mexico.; Table S4: Ancestral range estimation for each node.
